# Quality of life in patients with locally advanced head and neck squamous cell carcinoma undergoing concurrent chemoradiation with once‐a‐week versus once‐every‐3‐weeks cisplatin

**DOI:** 10.1002/cam4.4715

**Published:** 2022-03-28

**Authors:** Nandini S. Menon, Vanita Noronha, Vijay Maruti Patil, Amit Joshi, Atanu Bhattacharjee, Devanshi Kalra, Sarbani Ghosh Laskar, Vijayalakshmi Mathrudev, Kavita Nawale, Arati S. Bhelekar, Kumar Prabhash

**Affiliations:** ^1^ Department of Medical Oncology, Tata Memorial Centre Homi Bhabha National Institute Mumbai India; ^2^ Section of Biostatistics, Centre for Cancer Epidemiology, Advanced Centre for Treatment Research and Education in Cancer Navi Mumbai India; ^3^ Department of Radiation Oncology, Tata Memorial Centre Homi Bhabha National Institute Mumbai India

**Keywords:** chemoradiation, cisplatin, head and neck cancer, quality of life

## Abstract

**Introduction:**

This trial was conducted to compare the efficacy of low dose once‐a‐week cisplatin and once‐every‐3‐weeks cisplatin with radiation in locally advanced head and neck squamous cell carcinoma (LAHNSCC). The current analysis focuses on the quality of life (QoL) of patients in this trial.

**Methods:**

In this phase III randomized trial, patients with nonmetastatic LAHNSCC were randomized to receive cisplatin 30 mg/m^2^ once‐a‐week or 100 mg/m^2^ once every‐ 3‐weeks concurrently with radiotherapy. The primary endpoint was locoregional control. QoL was a key secondary endpoint. QoL was assessed using EORTC QLQ‐C30 and QLQ‐H&N35. QoL data were assessed at baseline, days 22 and 43 during treatment; and at 6, 12, 24 months. The linear mixed‐effects model was used for longitudinal analysis of QoL to determine the impact of treatment (arm) and time on QoL.

**Results:**

Three hundred patients were enrolled, data of 150 patients with available baseline QoL were analyzed. There was no significant difference in the global health status/QoL of the two treatment arms (*p* = 0.8664). There was no significant difference in the longitudinal QoL scores between the two treatment arms in all scales except constipation (*p* = 0.0096), less sexuality (*p* = 0.0002,), and financial difficulty (*p* = 0.0219). There was a worsening of the QoL scores in all scales in both arms during treatment, which improved after treatment completion in most scales.

**Conclusion:**

The use of once‐every‐3‐weeks cisplatin did not adversely impact QoL as compared to once‐a‐week cisplatin in combination with radiotherapy in LAHNSCC.

## Graphical abstract

Chemoradiation with once‐every‐3‐weeks cisplatin led to a significant improvement in locoregional control and a trend towards improved overall survival without significant worsening of QoL as compared to the once‐a‐week cisplatin regimen. The QoL scores worsened during treatment in most scales and improved after treatment completion, with a similar trend in both arms. Hence, once‐every‐3‐weeks cisplatin should be the treatment of choice in fit patients with LAHNSCC undergoing CRT.

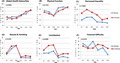

## 1 INTRODUCTION

Chemoradiotherapy (CRT) with cisplatin 100 mg/m^2^ given once‐every‐3‐weeks is the standard of care in locally advanced head and neck squamous cell cancer (LAHNSCC).^1^, ^2^ Often weekly cisplatin is substituted for once‐every‐3‐weeks cisplatin as it is easily administered, less toxic, and requires less supportive care.^3^

Acute toxicities of head and neck CRT such as mucositis, dysphagia, dysgeusia, and dermatitis adversely impact QoL.^4^ Xerostomia is the most common late toxicity of CRT, which leads to oral discomfort, impaired taste, problems with speech and swallowing, and poor oro‐dental hygiene.^5^, ^6^ Other late effects of CRT are hoarseness, subcutaneous fibrosis, chronic dysphagia due to mucosal atrophy, and increased risk of aspiration. Head and neck cancer and its treatment have a significant impact on the patients' QoL in both the disease‐specific health‐related QoL domains such as speech, salivary, and swallowing functions as well as general QoL domains such as physical, mental, emotional, and social health.^7^, ^8^, ^9^, ^10^, ^11^, ^12^, ^13^, ^14^

High‐dose cisplatin administered once‐every‐3‐weeks leads to significantly higher toxicity than lower dose cisplatin administered once a week. This toxicity may detract from the QoL of the patient. This trial was conducted to compare the efficacy of the two regimens, with the primary endpoint of locoregional control (LRC).^15^ The current analysis focuses on the QoL of patients in this trial.

## 2 PATIENTS AND METHODS

### 2.1 Study design and eligibility

This phase III randomized trial assessed the non‐inferiority of cisplatin 30 mg/m^2^ given once‐a‐week compared with cisplatin 100 mg/m^2^ given once‐every‐3‐weeks; both administered concurrently with curative intent radiotherapy in patients with LAHNSCC. The study was conducted at Tata Memorial Center, Mumbai, India. Patients with LAHNSCC with a primary in the oral cavity, oropharynx, hypopharynx, larynx, or metastatic cervical lymphadenopathy of unknown primary and planned for curative CRT, either adjuvant for one or more high‐risk features or definitive CRT for unresectable disease or organ preservation were included. All patients provided written informed consent. The study was approved by the institutional ethics committee and was conducted according to the principles laid down by the International Conference on Harmonization Good Clinical Practice guidelines, the Declaration of Helsinki, Schedule Y (Drugs and Cosmetic Act, 1940), and the Indian Council of Medical Research guidelines. The trial was registered at Clinical Trials Registry–India (identifier: CTRI/2012/10/ 003062) and funded by the Tata Memorial Centre Research Administration Council (TRAC).

### 2.2 Study treatment

Three hundred patients were randomly assigned 1:1 to cisplatin 30 mg/m^2^ once a week or cisplatin 100 mg/m^2^ once‐every‐3‐weeks (on days 1, 22, and 43) with curative radiotherapy.

### 2.3 Study end‐points

The primary endpoint of the study was to determine whether once‐a‐week cisplatin was non‐inferior to once‐every‐3‐weeks cisplatin in improving locoregional control (LRC). QoL was a key secondary endpoint. This analysis aimed to determine whether there was a difference in the QoL in patients who received once‐a‐week cisplatin as compared to those who received the once‐every‐3‐weeks cisplatin.

### 2.4 Quality of life assessment

QoL data were collected at baseline, on days 22 and 43 during CRT, and at 6,12, and 24‐months during follow‐up in patients without disease recurrence. QoL was assessed using the European Organization for Research and Treatment of Cancer Quality of Life Questionnaire C30 (EORTC QLQ‐C30) version 3.0 and EORTC QLQ Head and Neck Cancer‐Specific Module (QLQ H&N35) version 1.0. The QoL questionnaires were included in the analysis if they were self‐administered and had the date written properly within the time window for that time point. The questionnaires were administered in English, Hindi, and Marathi (validated translations were used).

The QLQ‐C30 has 30 questions from which five function scales, nine symptom scales, and one global health status/QoL scale are derived. The QLQ‐H&N35 has 35 questions from which 18 symptom scales are derived. The scores for all scales were calculated according to the EORTC Scoring Manual^16^ and range from 0 to 100. Higher scores in the function scales and global health status scales indicate a higher level of functioning and a better QoL respectively; while higher scores in symptom scales indicate more severe symptoms.

### 2.5 Statistical methods

Descriptive statistics were used to summarize demographic and clinical variables. QoL compliance was calculated as the percentage of assessable questionnaires to the total number of expected questionnaires (i.e., all patients alive at that time point) per time window. The linear mixed‐effects model was used for longitudinal analysis of QoL domains to determine the impact of treatment (arm) and time on QoL scores. The effect size (Cohen D) was calculated for the function and symptom scales.

### 2.6 Handling of missing data

Only patients with available baseline questionnaires were included in this analysis. The mean and standard deviation representation were carried from available cases. No data imputation were performed. Listwise deletion was not performed during this analysis. We considered the data for the visits where it was available and ignored the visits for which there were no data. During the statistical inference, the fixed effects parameter related to missing values was deleted to preserve the estimability. The missing level combinations for the random‐effect parameters were not deleted because it is possible to estimate the linear combinations of the random‐effect parameters. All statistical analysis was performed using the R Statistical Software version 3.4.0.

## 3 RESULTS

### 3.1 Summary of clinical data

Three hundred patients were randomized in this trial, 150 in each arm. The cumulative 2‐year LRC in the once‐every‐3‐weeks arm was significantly better than the once‐a‐week arm (73.1% vs. 58.5%, *p* = 0.014; HR, 1.76 [95%CI, 1.11 to 2.79]). Acute toxicities (84.6% vs. 71.6%, *p* = 0.006) and hospitalization for toxicity (31.1% vs. 13.3%, *p* < 0.001) were significantly higher in patients in the once‐every‐3‐weeks cisplatin arm as compared to those in the once‐a‐week cisplatin arm.

### 3.2 QoL compliance

**FIGURE 1:**
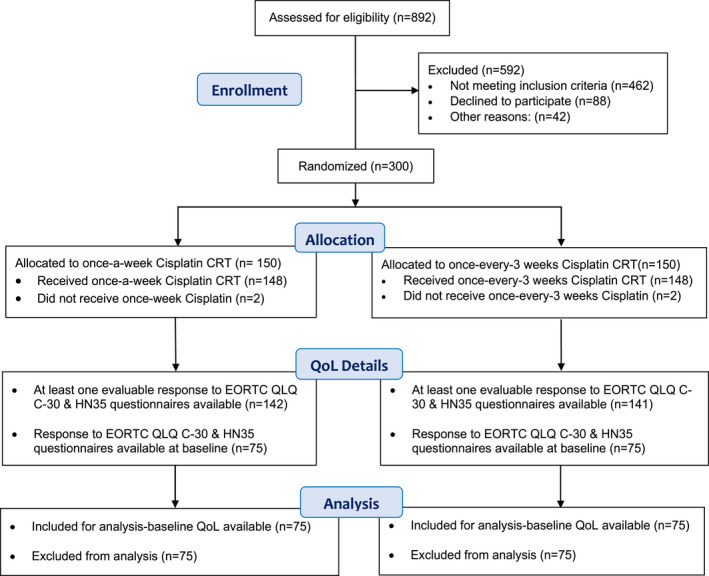
CONSORT diagram

QoL data that is, at least one assessable questionnaire was available for 283 patients. Baseline QoL data were missing for 150 patients and these patients were excluded from this analysis even if QoL data were available for subsequent visits (Figure 1). Visit wise dropout rates between the arms were compared and no‐significant difference were observed between the arms. The QoL compliance is shown in Table 1.

**TABLE 1:** Details of compliance with QoL questionnaires

Time	Once‐every‐3‐weeks cisplatin		Once‐every‐week cisplatin		T‐Test
	(*n* = 150)		(*n* = 150)		
	Number of eligible patients^a^	Questionnaires filled	Number of eligible patients^a^	Questionnaires filled	
		Number (%)^b^		Number (%)^b^	*p*‐value
Baseline	150	75 (50)	150	75 (50)	1.000
6 months	114	46 (40.35)	102	47 (46.07)	0.412
12 months	78	35 (44.87)	67	33 (49.25)	0.6202
24 months	35	19 (54.28)	35	11 (31.42)	0.090

^a^ Number of patients without locoregional recurrence at each time point, this was considered as denominator as locoregional control was the primary end point of this trial.

^b^ Only patients that had filled out the baseline questionnaires were included for analysis.

### 3.3 Baseline QoL assessment

There was no significant difference in the mean baseline QoL scores between the two arms in all the scales of the QLQ C‐30. Among the 18 symptom scales of H&N 35, there was a difference with moderate effect only in the scale for decreased sexuality (Table 3). Tables 2 and 3 show the baseline QoL scores.

**TABLE 2:** Baseline QLQ C‐30 scores

QoL scale	Once‐every‐3‐weeks cisplatin	Once‐every‐week cisplatin	Difference in mean scores	Effect size
	Mean score (SD)	Mean score (SD)		
Global health status/QOL	63.67 (34.15)	57.22 (37.85)	6.4	0.1788
Function scales				
Physical function	84.44 (16.45)	87.47 (14.45)	3.0222	0.1952
Role function	85.56 (24.55)	85.56 (23.62)	0	0
Emotional function	78.11 (22.04)	77.33 (24.22)	0.7778	0.0336
Cognitive function	87.33 (19.34)	90.22 (17.77)	2.8889	0.1555
Social function	80.00 (23.25)	74.89 (32.46)	5.1111	0.1810
Symptom scales				
Fatigue	25.92 (23.56)	22.96 (23.34)	2.9630	0.1263
Nausea & Vomiting	11.11 (16.74)	8.44 (15.82)	2.6667	0.1637
Pain	20.44 (26.36)	19.33 (23.25)	1.1111	0.0447
Dyspnoea	6.67 (17.33)	5.78 (16.77)	0.8889	0.0521
Insomnia	17.78 (30.67)	18.22 (25.28)	0.4444	0.0158
Appetite loss	27.11 (35.38)	21.33 (26.07)	5.7778	0.1859
Constipation	17.33 (28.13)	11.56 (22.92)	5.7778	0.2252
Diarrhea	6.67 (15.49)	5.78 (12.70)	0.8889	0.0627
Financial	36.44 (35.16)	43.56 (37.56)	7.1111	0.1955

**TABLE 3:** Baseline scores QLQ H&N‐35

QoL scale	Once‐every‐3‐weeks cisplatin	Once‐every‐week cisplatin	Difference in mean scores	Effect size
	Mean score (SD)	Mean score (SD)		
Pain	19.44 (20.01)	14.67 (18.32)	4.7778	0.249
Swallowing	22.11 (22.98)	19.22 (26.91)	2.8889	0.1154
Problems with senses	12.67 (21.89)	11.33 (20.71)	1.3333	0.0626
Speech problems	26.37 (24.08)	24.29 (26.32)	2.0741	0.0822
Trouble with social eating	22.33 (25.49)	19.78 (24.19)	2.5556	0.1028
Trouble with social contact	19.73 (23.64)	14.04 (19.06)	5.6889	0.2649
Less sexuality	23.11 (32.87)	10.22 (17.94)	12.8889	0.4868
Teeth	16.44 (27.05)	20.89 (31.37)	4.4444	0.1517
Mouth opening	27.56 (28.67)	27.11 (31.80)	0.4444	0.0147
Dry mouth	20.44 (23.82)	18.22 (25.87)	2.2222	0.0894
Sticky saliva	28 (29.53)	26.67 (33.33)	1.3333	0.0423
Coughing	16.89 (25.33)	16.44 (25.92)	0.4444	0.0173
Felt Ill	16 (24.73)	22.67 (33.40)	6.6667	0.2268
Use of pain killers	12.89 (16.34)	13.78 (16.52)	0.8889	0.0541
Need for nutritional supplements	8 (14.33)	7.56 (14.04)	0.4444	0.0313
Need for feeding tube	13.33 (16.44)	14.22 (16.59)	0.8889	0.0538
Weight loss	17.33 (16.76)	14.22 (16.59)	3.1111	0.1865
Weight gain	7.56 (14.05)	8.89 (14.84)	1.3333	0.0923

### 3.4 Change of QoL as a function of time

There was no significant difference in the global health status of the two treatment arms (*p* = 0.8664). There was no significant difference in the longitudinal QoL scores between the two treatment arms in all the functional and symptom scales except constipation (*p* = 0.0096), decreased sexuality (*p* = 0.0002), and financial difficulty (*p* = 0.0219). Overall, there was a worsening of QoL scores in all the scales in both arms during treatment (Table 4).

- Global health status and function scales

**TABLE 4:** Linear mixed effects model showing the impact of time and treatment arm on QOL scores

QoL scale	Time (*p* value)^b^	Arm (*p* value)^b^	Arm^a^ time
Global health status	0.0007^a^	0.8664	0.8924
Physical function	0.001^a^	0.1164	0.9325
Role function	0.016^a^	0.3549	0.7582
Emotional function	0.0008^a^	0.6037	0.3534
Social function	0.0066^a^	0.0868	0.1597
Cognitive function	0.1696	0.1765	0.2386
Nausea & vomiting	0.0001^a^	0.2444	0.9659
Fatigue	<0.0001^a^	0.6038	0.3152
Pain	0.0067^a^	0.2087	0.3009
Appetite loss	0.0002^a^	0.8304	0.6705
Insomnia	0.0011^a^	0.3208	0.3882
Diarrhea	0.0376^a^	0.1541	0.9267
Constipation	0.0169^a^	0.0096^a^	0.7525
Financial difficulty	0.0005^a^	0.0219^a^	0.0051
Pain (HN‐35)	0.0001^a^	0.286	0.7031
Swallowing difficulty	<0.0001^a^	0.4995	0.6662
Problems with Senses	0.1067^a^	0.5259	0.5779
Speech problems	0.0002^a^	0.1295	0.8544
Trouble with social eating	0.0015^a^	0.5407	0.6564
Trouble with social contact	0.0018^a^	0.072	0.7151
Decreased sexuality	0.0839	0.0002^a^	0.4742
Problems with teeth	0.042^a^	0.0552	0.0925
Decreased mouth opening	0.0054^a^	0.7641	0.377
Dry mouth	0.0953	0.93	0.6808
Sticky saliva	0.0093^a^	0.3947	0.9404
Cough	0.0053^a^	0.0784	0.6513
Felt Ill	0.0001^a^	0.2164	0.0274
Use of painkillers	<0.0001^a^	0.7649	0.6111
Need for a feeding tube	<0.0001^a^	0.5372	0.8493
Need for nutritional supplements	0.0611	0.9739	0.596
Weight loss	<0.0001^a^	0.5593	0.9265
Weight gain	0.002^a^	0.7845	0.3794

^a^ A significant difference was noted in the QoL scores in these scales.

^b^ A *p* value < 0.05 was considered significant.

Although there were no significant differences in the scores for the global health status and physical, social, emotional, role, and cognitive functioning in the linear mixed model in the two treatment arms; there was a significant difference in the scores over time. The global health status and function scores were lower during treatment and improved steadily after treatment completion up to 1 year and plateaued thereafter in both arms.

- Symptom scales

i) Nausea & vomiting

**FIGURE 2:**
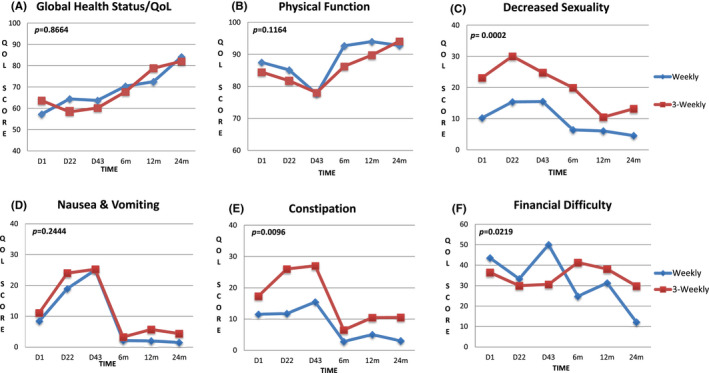
QoL scores in the various scales of the EORTC QLQ‐C30 and H&N 35. (A): Global Health status, (B): Physical Function, (C–F): Symptom scales for decreased sexuality, nausea & vomiting, constipation, and financial difficulty respectively. D1‐ day 1, D22‐ day 22, D 43‐ day 43, 6 m‐ 6 months, 12 m‐ 12 months, 24 m‐ 24 months. *p values* for the difference between the treatment arms from the linear mixed model

In patients included in this analysis, the incidence of grade 3 vomiting was 2.6% and 5.3% in the once‐a‐week and once‐every‐3‐weeks cisplatin arm, respectively *(p* = 0.442). The QoL symptom scores for nausea and vomiting progressively increased during CRT with the worst scores seen at day 43 in both arms. The scores were marginally higher in the once‐every‐3‐weeks cisplatin arm as compared to the weekly cisplatin arm, but this was not significant (*p* = 0.244, Figure 2).

ii) Constipation

The scores for constipation increased during treatment, peaked at day 43 in both arms, and declined steeply thereafter (Figure 2). Though the trend was similar in both arms, the scores for the once‐every‐3‐weeks cisplatin arm were significantly higher than that of the once‐a‐week cisplatin arm throughout (*p* = 0.0096).

iii) Sexuality

The scores for decreased sexuality were significantly higher in the once‐every‐3‐weeks cisplatin arm in comparison with the once‐every‐week cisplatin arm throughout treatment and follow‐up (*p* = 0.0002, Figure 2). There was a significant difference in the baseline scores between the two arms with a moderate effect size (Cohen D = 0.4868, Table 3). The time trend of the scores for decreased sexuality was similar to other symptom scales and was not significant (*p* = 0.0839, Table 4). The number of patients who responded to the questions on sexuality was similar in both arms at baseline and all subsequent time points, except at day 43 and 2 years. More patients responded to the questions in the once‐every‐3‐weeks arm as compared to the once‐a‐week arm at day 42 and 2 years, this difference was not statistically significant (*p* = 0.18). The difference at 2 years can be explained as the number of patients with disease recurrence was higher in the once‐a‐week arm while the difference at day 43 is not explained.

iv) Financial difficulty

There was a significant difference between the scores of the two arms for the financial difficulty scale (*p* = 0.0219). There was also a significant change in the financial difficulty scores over time in both arms (*p* = 0.0005). The scores were higher in the once‐a‐week cisplatin arm during treatment (worst score at day 43) and higher in the once‐every‐3‐weeks arm after treatment (worst score at 6 months) (Figure 2).

- Other symptom scales

The QoL scores for symptom scales––fatigue, pain, head & neck pain, difficulty in swallowing, loss of appetite, dyspnoea, diarrhea, cough, need for a feeding tube, use of pain killers, and problems with speech, senses, social contact, social eating were higher during treatment, improved after treatment completion. The scores for dry mouth increased during treatment and continued to remain slightly higher than baseline scores beyond 1 year. The difference in scores for dry mouth between the two arms was not significant (*p* = 0.0953).

## 4 DISCUSSION

In this study, QoL was assessed in patients with LAHNSCC during and after treatment with radiotherapy with once‐a‐week versus once‐every‐3‐weeks‐cisplatin. Longitudinal analysis using the linear mixed model showed that there was no difference in the global health status between the two arms. The QoL scores declined during treatment in most symptoms scales and improved after treatment completion in both arms. In most scales, the worst scores were seen on day 22 or 43 in both arms. Scores in most symptom scales reached baseline or were better than the baseline scores at 6 months except for social contact, social eating, problems with the senses, feeling ill, dry mouth, and difficulty in mouth opening. At 1‐year, the scores for all symptom scales except for dry mouth were better than baseline scores. This can be explained by the fact that xerostomia is a late effect of radiation and symptoms become more apparent with time.

A similar trend was seen in the study by Curran et al. in patients receiving radiotherapy alone or radiotherapy with cetuximab, where the QoL scores improved at 8 and 12 months and returned to baseline scores by 12 months.^9^ Karimi et al. also reported that patients with head and neck cancer receiving IMRT had a significant deterioration in the global health status and all the function scales during radiation which improved significantly at 3 months. The scores at 3 months were similar to baseline scores.^17^A study evaluating longitudinal changes in QoL showed that the difference in the overall QoL scores measured by the QLQ C‐30 was not statistically or clinically different at baseline and 2 years after adjuvant radiation. The scores for dry mouth, sticky saliva, and problems with social eating were worse at 2 years and this was both statistically and clinically significant. The problems with senses were clinically but not statistically worse and there was no difference in the other domains of H&N 35.^18^ This was similar to the pattern seen in our study.

There was no significant difference in the baseline scores in both the arms in all scales except sexuality. The reasons for the higher scores at baseline in the decreased sexuality scale in the once‐every‐3‐weeks arm is unclear. The scores in the once‐every‐3‐weeks arm remained consistently higher than the scores in the weekly arm throughout treatment and follow‐up. In the linear mixed model, the change in QoL scores over time was not statistically significant (*p* = 0.0839); while the difference of scores between the two arms was significant (*p* = 0.0002). These findings cannot be attributed to the treatment arm alone, hence we looked for other factors which could explain the difference. Patients are often uncomfortable about answering questions related to sexuality and missing data can skew results. We analyzed the number of patients in both arms who responded to the questions related to sexuality and found that the differences in the number of patients who responded at various time points were not statistically significant (*p* = 0.18). The number of patients who responded to the questions at baseline were the same in both arms which does not explain the difference in the baseline scores.

In this study, there was a significant difference in the financial difficulty scores of the two treatment arms. In contrast to other studies,^17^ the scores for the financial difficulty scale in the once‐every 3‐weeks arm did not deteriorate during chemoradiation but were higher at 6 and 12 months, and improved at 2 years. The patients in the once‐every‐3‐weeks arm had a higher incidence of acute toxicities and required more supportive care. Presuming that this would increase the costs of treatment, we expected higher scores in the once‐every‐3‐weeks arm. It is not clear why the financial difficulty scores were higher in the follow‐up period in the once‐every‐3‐weeks arm and whether long‐term toxicities (e.g., metabolic disorders) contributed to higher financial toxicity. The other possibility is that the cost incurred by the patients during treatment negatively impacted their finances (e.g., savings) and this was reflected in the financial difficulty scores after treatment. The scores for financial difficulty in the once‐a‐week arm worsened during CRT and improved after treatment, a similar trend was also reported by Karimi et al.^17^

Egestad et al. analyzed the health‐related QoL deterioration due to costs of treatment in patients with head and neck cancer undergoing radiation in Norway using the EORTC QLQ C‐30. The scores for the financial difficulty scale reported by them were in the lower range (mean 15–25), indicating limited financial difficulties which did not change significantly during the treatment period.^19^ In comparison, the mean scores for financial difficulty were higher in our study ranging from 29.8 to 41.3 in the once‐every‐3‐weeks arm and 12.1 to 50 in the weekly arm. This contrast in financial difficulty scores may be attributed to the difference in the health care systems in the two countries. Norway has a public‐funded healthcare system where citizens do not pay for health insurance and have equal access to health care.^19^ It also has a social security system where patients receive full pay while they are ill, and patients and their families have access to compensatory payments or social welfare assistance^19^; minimizing individual responsibility for the cost of medical care.^19^, ^20^ In India, the majority of the population does not have health insurance, and treatment costs are borne by the patient.^21^, ^22^ Various government policies and public insurance schemes available in India do not fully cover the cost of cancer therapy for all patients. The mean out of pocket expenditure for treatment is higher for cancer patients as compared to patients with other chronic diseases; both in the public and private sector.^22^ In India patients with cancer also spend more on nonmedical expenses (e.g., transport, food, lodging) as compared to other chronic diseases.^22^ This is especially relevant in our setting where most of the patients being treated at our center come from outside the city of Mumbai. The inability of cancer patients and their caregivers to work while on treatment leads to loss in family income. The per person loss in income for a cancer affected household in India is up to four times higher than that of a non‐cancer affected household.^22^ Hence, a large number of patients borrow money or resort to selling their assets to fund their treatment.^22^

An important factor in QoL analysis is the handling of missing data. Patients with missing baseline QoL questionnaires that is, 50% were excluded from the analysis. Of those included, the compliance with completion of QoL questionnaires declined with time in both arms, but the difference was not statistically significant (Table 1). Worse compliance in the once‐a‐week arm was probably due to a higher number of patients with disease recurrence in this arm.

Previous studies comparing various cisplatin schedules for concurrent CRT in head and neck cancer have focused mainly on clinical outcomes and toxicity.^1^, ^2^, ^23^ Few studies have reported on the impact on QoL with different cisplatin schedules and the improvement in QoL with weekly cisplatin has not been established. The ongoing NRG‐ HN009 study (NCT05050162) is a phase II/III trial comparing cisplatin 40 mg/m^2^ given weekly with 100 mg/m^2^ given once‐every‐3‐weeks.^24^ The results of this trial will provide information on whether low dose weekly cisplatin is truly less toxic as compared to high‐dose cisplatin and its impact on QoL.

In our study, despite higher acute toxicities, the once‐every‐3‐weeks cisplatin regimen led to a significant improvement in locoregional control and a trend toward improved overall survival without significant worsening of QoL as compared to the once‐a‐week cisplatin regimen.^15^ Hence, the once‐every‐3‐weeks cisplatin should be the treatment of choice in fit patients with LAHNSCC undergoing CRT.

## 5 LIMITATIONS

This was a single‐center study. Although 300 patients were enrolled in this trial, 50% (150 patients) did not have baseline QoL data and were excluded from the analysis. The significant amount of missing data is the main limitation of this study. The low participation rate limits the utility of interpretation of the reported results. In order to evaluate whether the different treatment regimens contributed to a differential pattern of dropout, we analyzed the visit‐wise dropout rates between the arms. We found no significant differences between the arms. The missing data suggest that systems need to be put in place to get more responses.

## 6 CONCLUSION

The use of once‐every‐3‐weeks cisplatin did not adversely impact QoL as compared to once‐a‐week cisplatin in combination with radiotherapy in LAHNSCC.

## CONFLICT OF INTERESTS

Vanita Noronha reports grants from Dr. Reddy's Laboratories, Amgen, and Sanofi, outside of the submitted work. Kumar Prabhash reports grants from Tata Memorial Center Research Administration Council, the Indian Cooperative Oncology Network, and Glenmark Pharmaceuticals, during the conduct of the study; grants from Dr. Reddy's Laboratories, Fresenius Kabi India, and Roche Holding, outside of the submitted work. All other authors declare no conflict of interest.

## AUTHOR CONTRIBUTIONS

Conceptualization: Nandini Menon, Vanita Noronha, and Kumar Prabhash. Methodology: Nandini Menon, Vanita Noronha, Vijay M Patil, Atanu Bhattacharjee, and Kumar Prabhash. Provision of study materials or patients: Nandini Menon, Vanita Noronha, Vijay M Patil, Amit Joshi, Devanshi Kalra, Vijayalakshmi Mathrudev, Kavita Nawale, Arati S. Bhelekar, and Kumar Prabhash. Investigation and data collection: Nandini Menon, Vanita Noronha, Amit Joshi, Vijay M Patil, Devanshi Kalra, Vijayalakshmi Mathrudev, Kavita Nawale, Arati S. Bhelekar, and Kumar Prabhash. Formal Analysis of Data: Nandini Menon, Atanu Bhattacharjee, Vijay M Patil, and Vanita Noronha. Administrative support: Nandini Menon, Vanita Noronha, Kumar Prabhash, Amit Joshi, Vijay M Patil, Vijayalakshmi Mathrudev, Arati Sanjay Bhelekar, Kavita Nawale, and Devanshi Kalra. Manuscript writing: Nandini Menon and Vanita Noronha. Final approval of manuscript: All authors. Accountable for all aspects of the work: All authors.

## CLINICAL TRIAL REGISTRATION

Clinical Trials Registry–India, Identifier: CTRI/2012/10/003062.

## PRECIS FOR USE IN THE TABLE OF CONTENTS

CRT with once‐every‐3‐weeks cisplatin did not adversely impact QoL as compared to once‐a‐week cisplatin in LAHNSCC.

## Supporting information


Appendix S1

## Data Availability

The data collected for the study (de‐identified individual participant data and a data dictionary defining each field in the set) will be made available on publication of this manuscript by the corresponding author (kumarprabhashtmh@gmail.com) for any scientific purpose with a signed data access agreement.
