# Supplementary material for: Quality of life in patients with locally advanced head and neck squamous cell carcinoma undergoing concurrent chemoradiation with once‐a‐week versus once‐every‐3‐weeks cisplatin
Source: Cancer Med. 2022 Mar 28;11(21):3939–48. doi: 10.1002/cam4.4715 (PMC9636500; doi:10.1002/cam4.4715)
Supplement: Supplementary file 1 — Appendix S1 [file CAM4-11-3939-s001.docx]

**SUPPLEMENTARY APPENDIX**

**Table 1: Effect Size (Cohen D) between the two arms at different time points**

| **QoL Scale** | **Effect size at baseline** | **Effect size at 6 months** | **Effect size at 12 months** | **Effect size at 24 months** |
| --- | --- | --- | --- | --- |
| **EORTC QLQ C-30** | | | | |
| Global Health Status/QoL | 0.1788 | 0.0952 | 0.2296 | 0.1123 |
| Physical Function | 0.1952 | 0.6094 | 0.499 | 0.1669 |
| Role Function | 0 | 0.1391 | 0.1707 | 0.5141 |
| Emotional Function | 0.0336 | 0.322 | 0.278 | 0.0181 |
| Cognitive Function | 0.1555 | 0.2615 | 0.0382 | 0.6522 |
| Social Function | 0.181 | 0.1725 | 0.0013 | 0.1711 |
| Fatigue | 0.1263 | 0.6597 | 0.4668 | 0.1781 |
| Nausea & Vomiting | 0.1637 | 0.1368 | 0.3166 | 0.2238 |
| Pain | 0.0447 | 0.1013 | 0.0028 | 0.3442 |
| Dyspnoea | 0.0521 | 0.1735 | 0.2774 | 0.8806 |
| Insomnia | 0.0158 | 0.0538 | 0.0804 | 0.181 |
| Appetite Loss | 0.1859 | 0.34 | 0.1029 | 0.0061 |
| Constipation | 0.2252 | 0.2565 | 0.2746 | 0.4493 |
| Diarrhoea | 0.0627 | 0.1275 | 0.4291 | 0.0462 |
| Financial Difficulty | 0.1955 | 0.4936 | 0.1976 | 0.5947 |
| **EORTC HN-35** | | | | |
| Pain | 0.249 | 0.066 | 0.153 | 0.0331 |
| Swallowing Difficulty | 0.1154 | 0.1608 | 0.4924 | 0.2513 |
| Problems with senses | 0.0626 | 0.5421 | 0.147 | 0.2155 |
| Speech Problems | 0.0822 | 0.4299 | 0.1368 | 0.2672 |
| Trouble with Social Eating | 0.1028 | 0.5083 | 0.1575 | 0.1164 |
| Trouble with Social Contact | 0.2649 | 0.7201 | 0.3096 | 0.185 |
| Decreased Sexuality | 0.4868 | 0.594 | 0.2805 | 0.5919 |
| Problems with Teeth | 0.1517 | 0.3239 | 0.0907 | 0.0076 |
| Decreased Mouth Opening | 0.0147 | 0.4847 | 0.0428 | 0.0425 |
| Dry Mouth | 0.0894 | 0.4824 | 0.1758 | 0.3193 |
| Sticky Saliva | 0.0423 | 0.3545 | 0.0989 | 0.1564 |
| Cough | 0.0173 | 0.1508 | 0.0876 | 0.355 |
| Felt Ill | 0.2268 | 0.5991 | 0.3292 | 0.0966 |
| Use of Painkillers | 0.0541 | 0.0624 | 0.1884 | 0.0694 |
| Need for Nutritional Supplements | 0.0313 | 0.0111 | 0.2129 | 0.5257 |
| Need for a Feeding Tube | 0.0538 | 0.1907 | 0.1884 | 0.4164 |
| Weight Loss | 0.1865 | 0.197 | 0.16 | 0.4253 |
| Weight Gain | 0.0923 | 0.2818 | 0.1669 | 0.139 |

**Table 2: Comparison of QoL Scores and Effect Size at 6, 12 & 24 months**

| **QoL Scale** | **At 6 months** | | | **At 12 months** | | | **At 24 months** | | | |
| --- | --- | --- | --- | --- | --- | --- | --- | --- | --- | --- |
|  | **Once-every- 3-weeks Cisplatin**  **Mean ± SD** | **Once-every-week Cisplatin**  **Mean ± SD** | **Effect Size** | **Once-every- 3-weeks Cisplatin**  **Mean ± SD** | **Once-every-week Cisplatin**  **Mean ± SD** | **Effect Size** | **Once-every- 3-weeks Cisplatin**  **Mean ± SD** | **Once-every-week Cisplatin**  **Mean ± SD** | **Effect Size** |  |
| Global health status/QoL | 67.75 ± 26.5 | 70.39± 28.8 | 0.0952 | 78.80 ± 20.04 | 72.47 ± 33.81 | 0.2296 | 82.02± 20.46 | 84.09±14.17 | 0.1123 |  |
| Physical Function | 86.23 ±11.01 | 92.62±9.95 | 0.6094 | 89.71 ± 9.34 | 93.94 ±7.52 | 0.4990 | 94.03 ± 7.66 | 92.73 | 0.1669 |  |
| Fatigue | 21.73 ± 16.38 | 11.35 ± 15.1 | 0.6597 | 19.68 ± 14.78 | 12.79±14.73 | 0.4668 | 17.54 ±20.39 | 14.14± 16.55 | 0.1781 |  |
| Nausea & Vomiting | 3.26 ±9.69 | 2.13± 6.6 | 0.1368 | 5.71 ± 15.62 | 2.02± 5.52 | 0.3166 | 4.38 ±15.56 | 1.51± 5.02 | 0.2238 |  |
| Pain (head & neck) | 12.86 ± 11.34 | 11.87±17.69 | 0.0660 | 10 ± 13.53 | 8.08±11.50 | 0.1530 | 8.77±13.46 | 8.33± 12.90 | 0.0331 |  |
| Difficulty in swallowing | 13.94± 16.85 | 11.34 ±15.48 | 0.1608 | 13.80 ± 13.69 | 7.82±10.40 | 0.4924 | 11.84±15.79 | 8.33 ± 9.86 | 0.2513 |  |
| Decreased sexuality | 19.93 ± 28.02 | 6.38 ±16.13 | 0.5940 | 10.47 ± 18.56 | 6.06± 12.37 | 0.2805 | 13.15 ± 16.27 | 4.54±10.78 | 0.5919 |  |
| Financial difficulty | 41.30 ± 35.96 | 24.82 ± 30.67 | 0.4936 | 38.09 ± 35.37 | 31.31±33.27 | 0.1976 | 29.82 ± 34.95 | 12.12 ±16.82 | 0.5947 |  |

**FIGURES**

**FIGURES QLQ C-30**

**Figure 1: QoL Scores for Role Function**

**Figure 2: QoL Scores for Emotional Function**

**Figure 3: QoL Scores for Social Function**

**Figure 4: QoL Scores for Cognitive Function**

**Figure 5: QoL Scores for Fatigue**

**Figure 6: QoL Scores for Pain**

**Figure 7: QoL Scores for Insomnia**

**Figure 8: QoL Scores for Loss of Appetite**

**Figure 9: QoL Scores for Dyspnea**

**Figure 10: QoL Scores for Diarrhea**

**FIGURES H&N 35**

**Figure 11: QoL Scores for Difficulty in Swallowing**

**Figure 12: QoL Scores for Pain (Head & Neck)**

**Figure 13: QoL Scores for Problems with Senses (Taste& Smell)**

**Figure 14: QoL Scores for Problems with Speech**

**Figure 15: QoL Scores for Trouble with Social Eating**

**Figure 16: QoL Scores for Trouble with Social Contact**

**Figure 17: QoL Scores for Difficulty in Opening Mouth**

**Figure 18: QoL Scores for Dry Mouth**

**Figure 19: QoL Scores for Sticky Saliva**

**Figure 20: QoL Scores for Cough**

**Figure 21: QoL Scores for Feeling Ill**

**Figure 22: QoL Scores for Painkiller Use**

**Figure 23: QoL Scores for Need of a Feeding Tube**

**Figure 24: QoL Scores for Use of Nutritional Supplements**

**Figure 25: QoL Scores for Weight Loss**

**Figure 26: QoL Scores for Problems with Teeth**
